# Supplementary material for: QTL identification and characterization of the recombination landscape of the mountain pine beetle (Dendroctonus ponderosae)
Source: G3 (Bethesda). 2025 May 7;15(7):jkaf101. doi: 10.1093/g3journal/jkaf101 (PMC12239636; doi:10.1093/g3journal/jkaf101)
Supplement: jkaf101_Supplementary_Data [file jkaf101_supplementary_data.pdf]

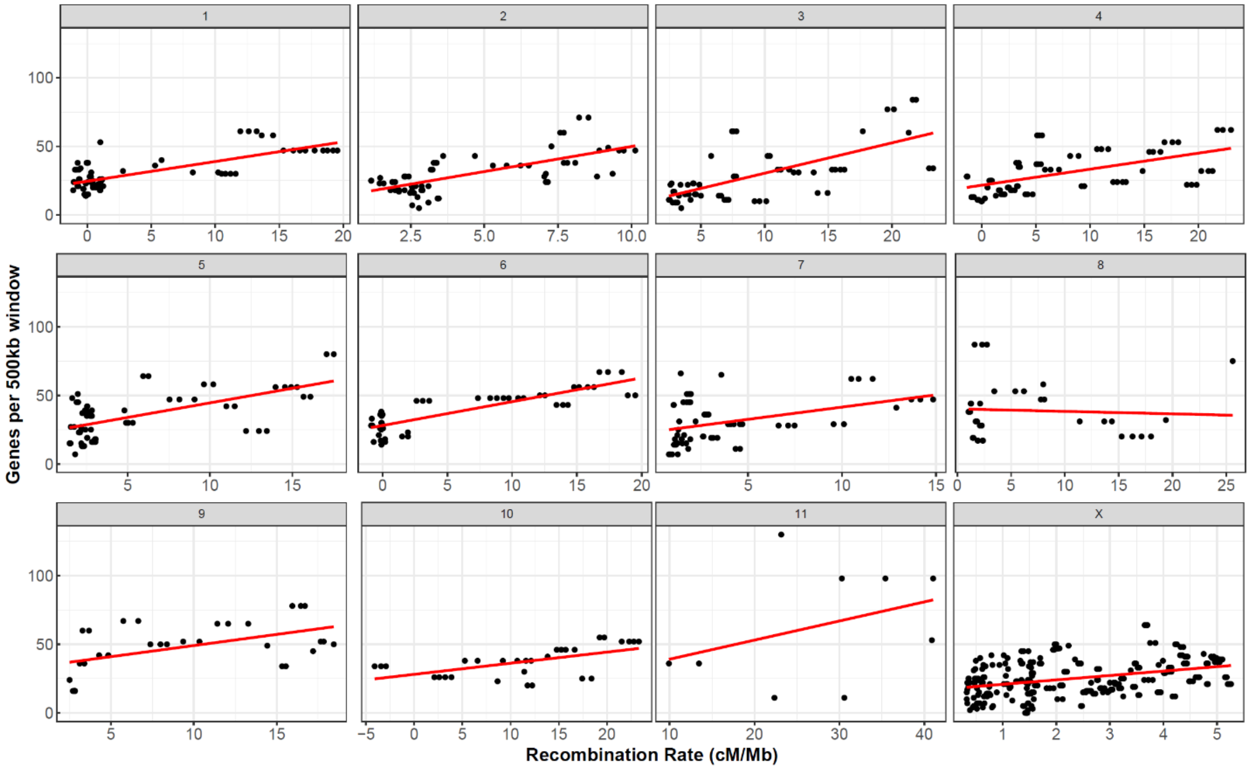

Supplemental Figure 1: Scatter plots comparing recombination rate and gene density, separated by chromosome. Linear regression for each chromosome is overlaid in red.
